# Hypokalemia-Induced Cardiac Arrest

**DOI:** 10.7759/cureus.35034

**Published:** 2023-02-15

**Authors:** Bradley Casey, Reese Hofstrand, Divyang Patel, Amol Bahekar, Alejandro Chapa-Rodriguez

**Affiliations:** 1 Internal Medicine, Cape Fear Valley Medical Center, Fayetteville, USA; 2 Cardiology, Cape Fear Valley Medical Center, Fayetteville, USA; 3 Critical Care Medicine, Cape Fear Valley Medical Center, Fayetteville, USA

**Keywords:** intubated, urine anion gap, cardiac arrest, hypokalemia, distal renal tubular acidosis

## Abstract

Renal tubular acidosis (RTA) refers to a group of disorders in which the elimination of hydrogen ions from the kidney or the reabsorption of filtered bicarbonate is impaired, resulting in metabolic acidosis. Hypokalemia is also prominent in different types of RTA. We are presenting an interesting case about a chronic alcoholic patient who presented to the emergency department and was found to be severely hypokalemic. During her hospital stay, she had multiple cardiac arrests likely secondary to her hypokalemia despite adequate treatment with potassium supplementation. We came to the conclusion of distal RTA in our patient based on hyperchloremic metabolic acidosis, sodium bicarbonate of 10 mmol/L, low potassium, blood urea nitrogen, and creatinine within normal limits, alkaline urine, and a positive urinary anion gap. It is likely that the cause of our patient's underlying type 1 RTA was secondary to her chronic alcohol abuse. Her potassium eventually returned to baseline, and she was discharged.

## Introduction

The kidneys are essential for preserving the body's acid-base balance, and this is achieved by reabsorbing filtered bicarbonate and eliminating extra hydrogen ions [1]. Renal tubular acidosis (RTA) is the collective term for renal conditions associated with malfunction in the elimination of bicarbonate or acid [1]. The human body functions optimally at a narrow pH range, and the kidneys are necessary to maintain that homeostasis [2]. Patients with distal renal tubular acidosis type 1 (RTA1) typically present with hyperchloremic metabolic acidosis, alkaline urine, elevated urine anion gap, and hypokalemia [3,4]. Almost a century has passed since hypokalemia and potassium depletion in the heart have been associated with cardiovascular diseases [5]. Plasma potassium levels are normally between 3.8 and 5.1 mmol/l, and deviations from that range (hypo- and hyperkalemia) increase the risk of cardiac arrhythmias [6]. Here we present an interesting case of a female that presented to the hospital with hypokalemia from RTA1, which resulted in multiple cardiac arrests.

## Case presentation

**Figure 1:**
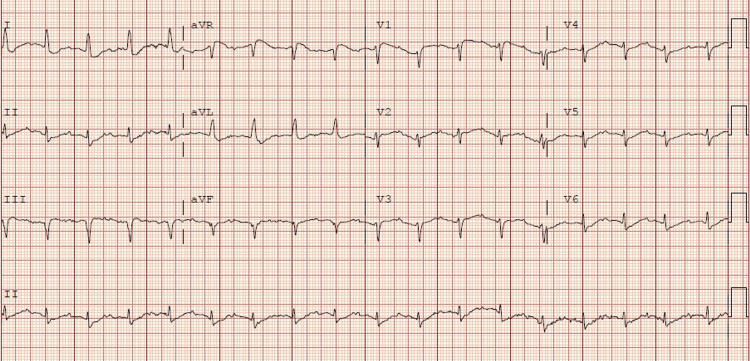
Electrocardiogram number 1: Sinus tachycardia rate of 105 beats per minute, narrow complex QRS, corrected QTC 556msec, Q waves in inferior leads

A 59-year-old female with a past medical history of chronic alcohol abuse, diastolic heart failure, depression, and hypertension presented to the emergency department with altered mental status. The patient’s husband reported that she had consumed more than 12 beers daily for 15 years. He reported that she has been binging with one bottle of vodka daily for the past one week, and he believed she has not consumed any alcohol for the past 24 hours. Husband confirmed she has not taken any of her home medications (semaglutide 0.25mg every seven days, levothyroxine 125mcg daily, pantoprazole 40mg daily, metoprolol succinate 50mg daily, amlodipine 10mg daily, amitriptyline 50mg daily, and atorvastatin 40mg daily) for at least two weeks. The patient presented to the hospital from home after her husband found her on the ground unresponsive for an unknown period of time. Bystanders performed CPR for an unknown amount of time before emergency medical services (EMS) arrived. When EMS arrived, a pulse was found and compressions were stopped. On the way to the emergency department, she was unresponsive to verbal and painful stimuli. When she arrived at the emergency department, she was intubated for airway protection. Initial blood work on arrival can be seen in Table 1. Initially, there was the concern of an infectious process due to high white blood cell count. Due to chronic alcohol consumption, hepatic encephalopathy was also on the differential as ammonia was elevated. Numerous differentials were considered at this time, including chronic alcoholism, poor oral intake, medication overdose from home medications, and over-the-counter herbal medication consumption. The patient uses a hometown pharmacy, and a voicemail was left for a callback so more information could be obtained on her home medications. Due to the severe hypokalemia and altered mental status, an electrocardiogram (EKG) was ordered (Figure 1), which showed a sinus tachycardia rate of 105 beats per minute, corrected QTC 556 msec, and Q waves in inferior leads. A femoral central line was placed, and the patient was started on 20 mEq/hr of potassium chloride. The ptient was then transferred to the ICU for the continuation of care.

**Table 1:** Patient's laboratory values

Laboratory Test	Reference Range	Patient's Lab
Pertinent complete blood count Results		
White Blood Cell Count	4.5 - 12.5 x10*3/µL	24.7x10*3/µL
Hemoglobin	12.0 - 16.0 g/dL	10.6 g/dL
Mean Corpuscular Volume	81.0 - 99.0 fL	104.5 fL
Platelets	150 - 450 x10*3/µL	219x10*3/µL
Pertinent Chemistry Panel Results		
Sodium	136 - 145 mmol/L	135 mmol/L
Potassium	3.5 - 5.1 mmol/L	1.2 mmol/L
Bicarbonate	21 -32 mmol/L	10 mmol/L
Chloride	98 - 107 mmol/L	117 mmol/L
Blood Urea Nitrogen	7 - 25mg/dL	18 mg/dL
Creatinine	0.60 mg/dL	1.30 mg/dL
Glucose	74 - 106 mg/dL	144 mg/dL
aspartate aminotransferase	15 - 37 U/L	81 U/L
alanine transaminase	12 - 78 U/L	53 U/L
glomerular filtration rate	>60.0 mL/min/1.73m*2	>60.0 mL/min/1.73m*2
Pertinent Arterial Blood Gas Results		
Arterial pH	7.35 - 7.45 pH	7.16 pH
Carbon Dioxide	35.0 - 45.0 mm Hg	44 mm Hg
Bicarbonate on Arterial Blood Gas	22.0 - 26.0 mEq/L	10.9 mEq/L
Arterial Potassium	3.50 - 5.30 mmol/L	<1.10 mmol/ L
Pertinent Urinalysis Results		
Urine Sodium	No reference range given	35 mmol/L
Urine Potassium	No reference range given	13 mmol/L
Urine Chloride	No reference range given	31 mmol/L
Urine Anion Gap	No reference range given	17 mmol/L
Urine Leukocytes	Negative	Negative
Urine Nitrites	Negative	Negative
Urine Bacteria	None Seen	None Seen
Urine White Blood Cell Counts	0-10 / high powered field	6 high powered field
Other Laboratory Value		
Ammonia	11 - 32 µmol/L	108 µmol/L
Thyroid Stimulating Hormone	0.358 - 3.740 µIU/mL	2.630 µIU/mL
Free T4	0.76 - 1.46 ng/dL	1.22 ng/dL
Ethanol Level	<3 mg/dL	<3 mg/dL

**Figure 2:**
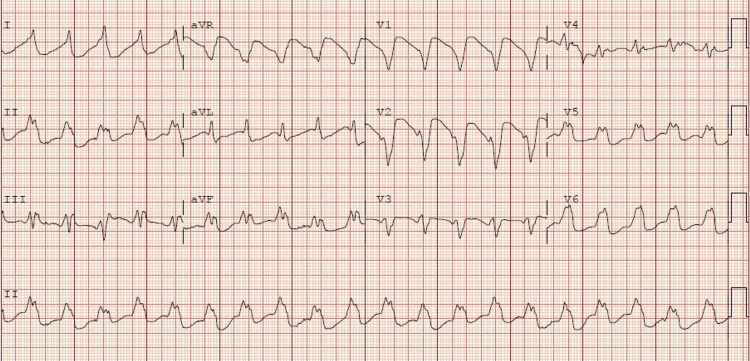
Electrocardiogram number 2 after the first cardiac arrest that showed wide complex tachycardia with intraventricular conduction delay likely secondary to underlying right bundle with the rate of 122 beats per minute

**Figure 3:**
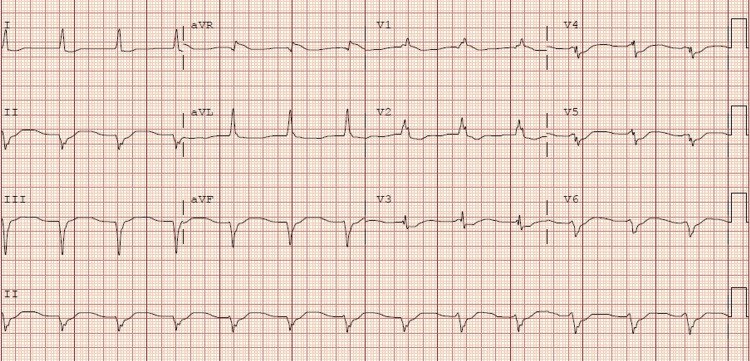
Electrocardiogram number 3: Accelerated junctional rhythm and right bundle branch block with a rate of 77 beats per minute

**Figure 4:**
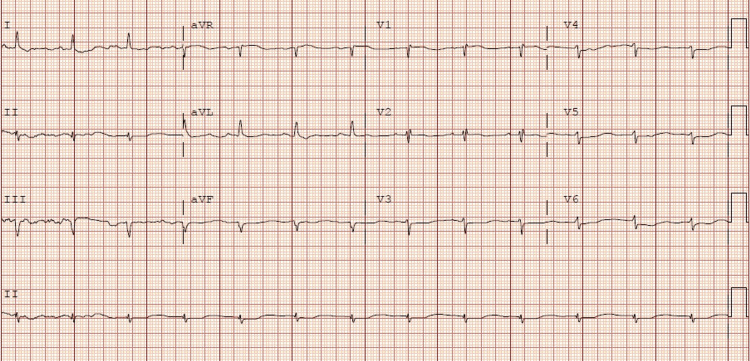
Electrocardiogram number 4: Narrow complex sinus rhythm 78 beats per minute

While in the ICU the patient started to have bradycardia and wide complex arrhythmia (no rhythm strips available). Staff was unable to palpate a pulse so advanced cardiac life support (ACLS) was initiated, and she received one round of ACLS before return of spontaneous circulation (ROSC) was achieved. During the first round of ACLS the patient received 50 mEq sodium bicarbonate, 1mg of epinephrine, and 2g of magnesium sulfate. Even though sodium bicarbonate is not part of ACLS protocol, it was given in this patient due to her home medications including a tricyclic antidepressant. Her husband endorsed no home medication intake for weeks but could not rule out cardiotoxicity from excessive tricyclic antidepressant intake. The patient used a local pharmacy that closed at 5:00pm; no after-hours answering service was available to help us determine the last time she had picked up her home medications. After ROSC was achieved the patient had another EKG. Labs were repeated, revealing magnesium serum levels within normal limits, and low potassium (Table 2). Second EKG after ROSC was achieved (Figure 2) showed wide complex tachycardia with intraventricular conduction delay likely secondary to the underlying right bundle with rate of 122 beats per minute. When wide complex arrhythmia was seen, another 2g of magnesium was given, and a repeat EKG was performed (Figure 3) which showed a rate of 77 beats per minute with accelerated junctional rhythm and right bundle branch block. The patient continued to receive 20 mEq/Hr of potassium chloride. Due to severe persistent hypokalemia (Table 2), there was concern that the patient may have RTA. Workup included urine PH of 8.0, and elevated urine anion gap (Table 1) suggesting of distal RTA. Approximately six hours after the first arrest she went into pulseless electrical activity (PEA) arrest without any signs of ventricular arrhythmia. She received another 50mEq of sodium bicarbonate, 1mg epinephrine, and 2g magnesium sulfate, and ROSC was achieved after 1 round of ACLS. Repeat potassium was low (Table 2) and repeat EKG (Figure 4) showing narrow complex sinus rhythm 78 beats per minute. Approximately nine hours after the second cardiac arrest the patient went into PEA arrest after becoming bradycardic (no rhythm strip available), and another round of ACLS was performed. During the third round of ACLS the patient again received 2g magnesium sulfate, 50mEq sodium bicarbonate, and 1mg epinephrine and ROSC was achieved at the next pulse check. The patient had an extensive ICU stay and was eventually extubated. The 20 mEq/hr of potassium was discontinued once her potassium returned to normal range (Table 2). When the patient was extubated, she reported that she was drinking more than 24 beers a day and a bottle of vodka. Patient did confirm that she had not taken any of her home medications for weeks prior. The patient was eventually discharged to a rehabilitation facility.

**Table 2:** Potassium values of the patient from the time she arrived until her potassium returned to a normal range. Included are also the potassium values and time that she went into cardiac arrest. From the time the patient arrived at 7:30am she received 20mEq/Hr of potassium through a central line until she returned to normal at the bottom of the chart 7:00pm

Time	Potassium value (Range 3.5 - 5.1 mmol/L)
7:30am (Arrival)	1.2
11:17am	1.5
6:00pm	1 (Cardiac Arrest number 1)
12:00am (Next day)	1.6 (Cardiac Arrest Number 2)
9:00am	1.8 (Cardiac Arrest Number 3)
1:00pm	2
7:00pm	2.2
1:00am (Next day)	2.8
3:00am	2.5
10:00am	3.1
6:00pm	3.2
7:00pm	3.9

## Discussion

Distal RTA1 was the first form of RTA described in the literature in 1935 [2]. RTA1 is a disorder that affects the distal nephron's ability to secrete protons in response to metabolic acidosis [3]. The kidneys are responsible for continuously restoring the systemic bicarbonate pool. This is done by reabsorbing the majority of bicarbonate that is filtered by the glomeruli and by generating new molecules through ammoniagenesis [3]. In the presence of systemic acidosis, healthy kidneys will try to increase acid secretion to lower urine pH. However, if you have normal GFR and no urinary tract infection, high urine pH (pH > 6) can indicate underlying RTA. One of the initial steps in diagnosing RTA is to rule out hyperchloremic metabolic acidosis secondary to chronic diarrhea (which is the most common cause of hyperchloremic acidosis) [4]. Hypokalemia is a common symptom of both distal and proximal RTA, while hyperkalemia is indicative of hyperkalemic RTA [4]. The composition of urine is important for the diagnosis of RTA. In metabolic acidosis, a patient with normal renal function and intact urinary acidification mechanisms will produce urine with a pH of ≤5.3 in order to restore the plasma pH [4].

The first step in diagnosing RTA is to look at serum PH and bicarbonate levels [1]. With RTA1, the bicarbonate level is normally less than 10 to 20 mEq/L, type 2 RTA has a bicarbonate level of 12 to 18 mEq/L, and type 4 has a bicarbonate level greater than 17 mEq/L [1]. The next step would be to look at the level of potassium in the blood, as types 1 and 2 are associated with hypokalemia and type 4 with hyperkalemia [1]. BUN and creatine should also be examined as acute kidney injury could be one of the underlying driving forces for the acidosis that is seen most prominently in type 1 and type 2 [1]. Next, we need to look at the urinalysis and urine electrolytes, as RTA1 will have inappropriately alkaline urine (greater than 5.5) despite metabolic acidosis [1]. Urine electrolytes are also important as they will help us calculate the urine anion gap ([sodium plus potassium] minus chloride) because a positive gap along with hyperchloremic metabolic acidosis is suggestive of RTA. We came to the conclusion of distal RTA in our patient based on the above. Our patient came in with hyperchloremic metabolic acidosis, sodium bicarbonate of 10 mmol/L, low potassium, blood urea nitrogen, and creatinine within normal limits, alkaline urine, and a positive urinary anion gap. It is likely that the cause of our patient's underlying RTA1 was secondary to her chronic alcohol abuse.

The treatment of RTA1 in our patient was difficult for many reasons, but her hypokalemia was the primary treatment target. Even though our patient received 20mEq/hour of potassium through a femoral central line, her potassium was still reported in the 1.0-2.0mmol/L range with cardiac arrhythmias. Alkali administration with sodium bicarbonate is the treatment of choice for RTA1 [1]. In her case, treatment was complicated by the risk of correcting the acidosis with bicarbonate and causing the potassium to shift intracellularly, which would worsen her already severe hypokalemia.

Combating sudden cardiac death has been a major challenge in medicine worldwide. Our understanding of sudden cardiac death involves a combination of structural abnormalities and environmental changes in the myocyte/Purkinje membranes [5]. In this particular case, the perturbing factor is hypokalemia, and it is also one of the common causes of sudden cardiac death [5]. Potassium is an abundant intracellular cation used to promote a charge gradient across the cellular membrane [6]. In a healthy person, homeostasis is regulated by ATPase pumps which utilize available potassium to maintain the charge gradient [6]. Normal serum potassium is 3.8-5.1 mmol/L and significant derangements in either direction can result in arrhythmias [6]. It is possible to have hypokalemia for a number of reasons, including decreased potassium intake, transcellular shifts (increased intracellular uptake), and increased potassium loss (skin, gastrointestinal, and renal) [7]. Hypokalemia promotes Q-T prolongation with a risk of torsades des pointes, which can lead to ventricular fibrillation and sudden cardiac death [6]. ECG findings in hypokalemia: 1) flattening or T-wave inversion in potassium 3.0-3.8 mmol/L; 2) Q-T prolongation, visible U-wave, mild ST depression (0.5mm), ectopic beats in potassium 2.3-3.0 mmol/L; 3) torsades de pointes, ventricular fibrillation in potassium <2.3 mmol/L [6]. This is likely why our patient had wide complex arrhythmia leading to her first cardiac arrest. You can also see the effect that the potassium level has on an EKG in Figure 2.

Treatment of severe hypokalemia in the acute setting is centered around minimizing potassium loss and replacing the potassium deficit [8]. Total body potassium deficit can be from 200 to 400 mEq for every 1 mEq decrease in serum levels [9]. Intravenous potassium is indicated when new arrhythmias begin or potassium decreases to <2.5 mmol/L [9]. The maximum dose of IV potassium is 20 mEq/h [9]. If the patient is having life-threatening arrhythmias in this setting, an even more rapid infusion of potassium may be necessary. An initial infusion of 2 mEq/min followed by 10 mEq IV over 5-10 minutes may be performed under continuous electrocardiogram to avoid cardiovascular collapse [9].

While treating severe hypokalemia is the first priority, attention should also be given to determining the root cause and treating that source when possible. RTA can be seen in patients with chronic alcoholism and can result in significant serum electrolyte abnormalities and acid-base disequilibrium [10]. We determined that chronic alcoholism was the root cause of our patient's RTA and electrolyte abnormalities.

## Conclusions

RTA1 is a medical condition that can cause serious electrolyte abnormalities, as were seen in our patient. Despite being treated with the maximum hourly dose of potassium allowed through a femoral central line, she continued to have cardiac arrhythmias. Hypokalemia is seen daily in hospitals across the nation, and RTA1 needs to be high on the differential. Severe hypokalemia needs to be monitored closely and its cause investigated promptly due to the increased risk of cardiac compromise.
